# Hospital acquired infections in Intensive Care Unit: A study on incidence, antibiotic resistance profile and outcome of the patients in a tertiary care unit in Eastern India

**DOI:** 10.3934/microbiol.2025025

**Published:** 2025-07-21

**Authors:** Mandira Chakraborty, Sayani Sardar, Debasish Ghosh, Biyanka Sau, Maria Teresa Mascellino, Arkit Ghoshal, Aniket Rout, Silpak Biswas, Anita Nandi Mitra

**Affiliations:** 1 Department of Microbiology, Calcutta Medical College and Hospital, Kolkata, India; 2 Critical Care Unit In-charge, Department of Anesthesiology, Calcutta Medical College, Kolkata, India; 3 Department of Public Health and Infectious Diseases, Sapienza University of Rome, Rome, Italy; 4 Department of Microbiology, School of Tropical Medicine, Kolkata, India

**Keywords:** hospital acquired infections, antibiotic resistance, antibiotic susceptibility testing, catheter-related bloodstream infection, ventilator-associated pneumonia

## Abstract

Hospital acquired infections (HAI) are the most common cause of mortality among critically ill patients because of various predisposing factors such as co-morbidities (medical or surgical), invasive devices, a long-term stay in the Intensive Care Unit (ICU), and the use of broad-spectrum empirical antibiotics. HAI in ICUs include mainly four types of infections: Catheter-related bloodstream infection (CRBSI), ventilator-associated pneumonia (VAP), catheter-related urinary tract infection (CAUTI), and surgical site infections (SSI). In this study, we aimed to characterise the bacteriological andantibiotic resistance profiles of all types of HAI along with their outcomes in the ICU of a tertiary care hospital in Eastern India. Patients included in this study were all critically ill patients aged above 12 years who had one or more devices inserted and were admitted in the ICU due to some medical or surgical complication for more than 48 hours. This was a prospective study for a period of three months. Appropriate specimens were collected from admitted patients suspected of having infections for identification and antibiotic susceptibility testing. The outcomes were determined based on either the discharge of the patient, a transfer to a separate ward, or death within the hospital. A total of 169 patients were included in the study, of which 65 patients (38%) acquired an HAI in the ICU. Thirteen patients were diagnosed with multiple types of infections. There were 72 device related infections, of which CRBSI made up 36%, VAP made up 23%, CAUTI made up 20%, and SSI made up 21% of the total patients. The most isolated organism in the ICU setup was *Klebsiella* spp. (35%), followed by *Enterococcus* spp. (22%). We found that 92% of the *Klebsiella* spp. was resistant to Carbapenem and 30% were Vancomycin-Resistant *Enterococcus* (VRE). The highest mortality was found associated with VAP (73%), followed by CRBSI (52%), SSI (40%), and CAUTI (31%) in the ICU setting. The findings of this study are of great clinical importance and will help in preventing and controlling the spread of HAIs in the ICU.

## 1. Introduction

Hospital acquired infections (HAI) are the most common cause of mortality, especially among critically ill patients. It has become a major public health burden [1],[2]. Each year, HAIs are responsible for more than 140,000 deaths worldwide [1],[2]. Moreover, HAIs inflate the cost of care due to prolonged hospital stays and the consumption of large amounts of antibiotics. Nosocomial infections in the Intensive Care Unit (ICU) can be explained by the epidemiological triad, which is a model that describes the interaction between three factors that can lead to disease: The agent, the host, and the environment. Host factors in the ICUs involve comorbidities (both medical and surgical), long-term stays in the ICU, invasive devices inserted during the ICU stay, immunosuppression, and the use of broad-spectrum empirical antibiotics, while agent's factors include the virulence of the causative organisms and its antimicrobial resistance [3]–[8]. The colonisation of different bacterial species on the hospital environment surfaces and medical equipment is the third factor responsible for HAIs [9],[10]. The patients admitted in the ICU acquire nosocomial infections either from their endogenous flora or from the hospital environment [11].

HAIs in the ICU include four types of infections: Three device-associated infections, namely catheter-related bloodstream infection (CRBSI), catheter-related urinary tract infection (CAUTI), and ventilator-associated pneumonia (VAP), and the fourth infection is surgical site infections (SSI) that occur in patients admitted to the ICU due to any other surgical complications [12]. VAP represents pneumonia that develops in patients on mechanical ventilation for more than 48 hours. 5–40% of patients may develop VAP following intubation [13]–[15]. A recent study showed that VAP in India occurred at a frequency of 9.7% [16].

CAUTI refers to significant bacteriuria in a patient with an indwelling urinary catheter for more than two consecutive days. It may be asymptomatic or symptomatic, with symptoms such as a fever, pain in the lower abdomen or back, and haematuria [17]. In the ICU, 95% of UTIs were found as CAUTIs [18],[19]. CRBSI is defined as the presence of bacteraemia that originate from an intravenous catheter. According to a few Indian studies, the prevalence of CRBSI was 27–56% [20]–[22]. The World Health Organization (WHO estimated that the average mortality among patients in the ICU was 52.3% between 2000–2018.

HAI types and frequency can vary with different hospitals and populations [23],[24].The aim of this study was to determine the incidence of HAIs in the ICU of a tertiary care hospital in Eastern India, characterise the bacteriological and antibiotic resistance profiles of all types of HAI, identify the most common infection associated with the use of devices in the ICU, and evaluate the outcome of the infected patients in terms of the length of the ICU stay and any associated mortality.

## 2. Materials and methods

### 2.1. Study population and sample collection

A prospective study was carried out from November 2022 to January 2023 on a total of 169 patients who were admitted to the ICs of Medical College and Hospital (MCH), a tertiary care hospital in Kolkata, India. All critically ill patients were above 12 years of age, had one or more devices inserted, and were admitted due to some medical or surgical complication. A thorough history and associated risk factors of these patients were taken. A total of 250 clinical specimens were collected: 93 blood specimens, which included peripheral (53) and central line blood (40); 86 urine specimens; 37 pus specimens from SSIs; 26 endotracheal aspirate specimens; and 8 sputum specimens. All clinical samples were collected by following a standard protocol [25] at the bedside. Then, the samples were transported to the Microbiology Laboratory of the Department of Microbiology, Medical College and Hospital, Kolkata, India, as soon as possible. The clinical specimens that were processed included endotracheal tube (ET) and tracheal aspirates, sputum, blood from central line catheter insertion sites, urine, and swabs from the surgical site incision. A time cut-off of 48 h after admission was conventionally used to differentiate between hospital and community acquired infections [26].

### 2.2. Bacterial culture

For the blood cultures, blood samples were aseptically collected and inoculated in BacT/ALERT bottles for the aerobic cultures. Positive signaled blood culture bottles were initially inoculated on blood and MacConkey agar and aerobically incubated at 37 °C overnight. The bacterial species were identified by the colony characteristics on the MacConkey and blood agar, Gram staining, motility, routine rapid tests such as catalase and oxidase, and routine biochemical tests such as indole, triple sugar iron agar, urease, and the citrate utilisation test. A further identification of the bacteria was performed using the automated VITEK 2 compact system (bioMerieux) according to manufacturer's instructions.

### 2.3. Antibiotic Susceptibility Test (AST)

Antibiotic susceptibility was measured on Mueller Hinton agar following Clinical and Laboratory Standards Institute (CLSI) guidelines [27]. A total of seven classes of antibiotics were tested for all microorganisms, including the following: (a) penicillins, such as ampicillin (10 µg); (b) beta-lactam-beta-lactamase inhibitor combination, such as piperacillin/tazobactam (100/10 µg) and amoxycillin/clavulanic acid (20/10 µg); (c) cephalosporins, such as cefepime (30 µg), cefotaxime (30 µg), cefuroxime (30 µg), ceftriaxone (30 µg), and ceftazidime (30 µg); (d) aminoglycosides, such as amikacin (30 µg) and gentamicin (10 µg); (e) carbapenems, such as imipenem (10 µg) and meropenem (10 µg); (f) fluoroquinolones, such as ciprofloxacin (5 µg) and levofloxacin (5 µg); and (g) trimethoprim/sulfamethoxazole (1.25/23.75 µg). Multidrug-resistant (MDR) strains were assessed for the antibiotic susceptibility using the automated VITEK 2 compact system (bioMerieux) according to manufacturer's instruction. The results were interpreted according to the CLSI criteria [27]. Additionally, vancomycin-resistant *Enterococcus* (VRE) was detected by both the disc diffusion assay and VITEK 2. To determine the presence of vancomycin-resistant Enterococcus, the disc diffusion method (disc content 30 µg) was utilized and performed on Mueller Hinton agar with a zone diameter of ≤14 mm, which was taken as VRE as per the CLSI guidelines [27]. Moreover, the results corroborated with those of the VITEK 2 compact system. The VITEK 2 GP ID & AST-P628 and VITEK 2 GN ID & AST-N280VITEK cards were used.

### 2.4. Data analysis

All patients' data were electronically stored in a relational database system that was specifically developed for the study. Then, the data were exported into a spreadsheet for further analyses. The prevalence of antibiotic resistance was estimated as the proportion of positive results over the entire study sample. MDR occurs when bacteria become resistant to at least one antibiotic in three or more antimicrobial categories.

## 3. Result

**Figure 1.**
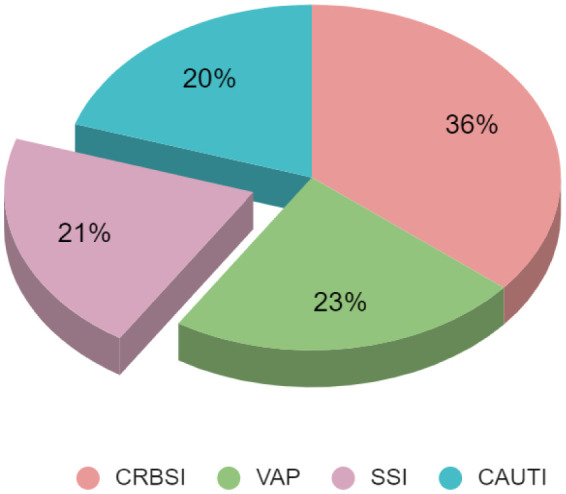
Distribution of different types of Hospital Acquired Infections in ICU found in this study. CRBSI = Catheter Related Bloodstream Infection; VAP = Ventilator Associated Pneumonia; CAUTI = Catheter Associated Urinary Tract Infections; SSI = surgical site infections.

A total of 169 patients admitted in the ICU were included over the three months in the study. Among them, 65 patients (38%) developed HAIs. Interestingly, male patients were more infected (65%) than female patients (35%). 13 patients got infections in multiple sites including the urinary tract, the respiratory tract, and the blood stream. There were 72 device related infections, of which CRBSI made up 36%, VAP made up 23%, CAUTI made up 20%, and SSI made up 21% of the total (Figure 1). Two patients developed pneumoniae after extubating.

**Figure 2.**
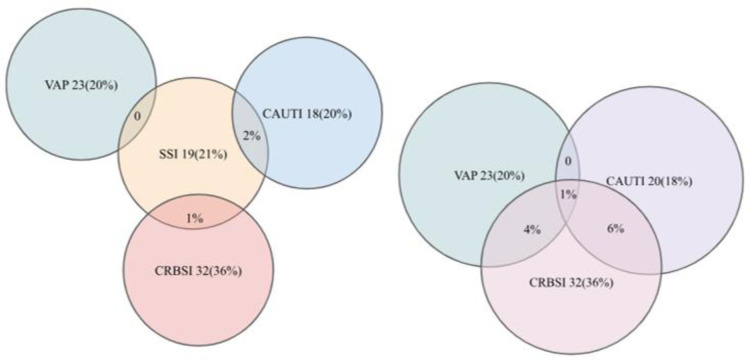
Venn diagram showing inter-relationship between the different device-associated infections.

*Klebsiella* spp. (35%) was the most isolated organism in the ICU from all types of nosocomial infections, except for the CAUTIs, where the most common pathogen was *Enterococcus* (27%) (Table 1). Figure 2 shows the inter-relationship between the different device-associated infections. *Klebsiella* spp. followed by *Acinetobacter baumanii* complex were the main causative organisms for VAP in our setting. Only 15% of the VAP cases were caused by *Pseudomonas aeruginosa*. Although most common organism causing CAUTI was *Enterococcus* spp. was the most common organism for the CAUTI cases, among Gram-negative organisms, *Klebsiella* was the most widespread, accounting for 22% of the infections, followed by the *Acinetobacter baumannii* complex (11%). Though community acquired UTI is most commonly caused by *Escherichia coli*, in catheterised patients admitted to the ICU, it only accounted for 6% of the total infections. *Klebsiella* spp. was found to be most frequently isolated Gram-negative bacteria for the CRBSI cases, accounting for 34 % of the cases. Among Gram-positive bacteria, *Enterococcus* was also isolated from 34% of the CRBSI cases. SSIs in an ICU setting is most frequently caused by *Klebsiella* spp. (32%), followed by *Escherichia coli* in 26% of the cases.

**Table 1.** Microorganism associated with different types of hospital acquired infections.

		Device related			SSI	Total
Microorganisms	Pneumonia n (%)	VAP n (%)	CAUTI n (%)	CRBSI n (%)	Surgical site infection n (%)	n (%)
*Escherichia coli*	0	1 (5)	1 (6)	2 (6)	5 (26)	9 (10)
*Klebsiella* spp.	1 (50)	10 (50)	4 (22)	11 (34)	6 (32)	32 (35)
*Acinetobacter baumannii* complex	1 (50)	6 (30)	2 (11)	7 (22)	0	16 (16)
*Pseudomonas* spp.	0	3 (15)	3 (17)	1 (3)	4 (21)	11 (12)
*Enterococcus* spp.	0	0	5 (27)	11 (34)	4 (21)	20 (22)
*Candida* spp.	0	0	3 (17)	0	0	3 (3)
Total	2 (100)	20 (100)	18 (100)	32 (100)	19 (100)	91 (100)

**Figure 3.**
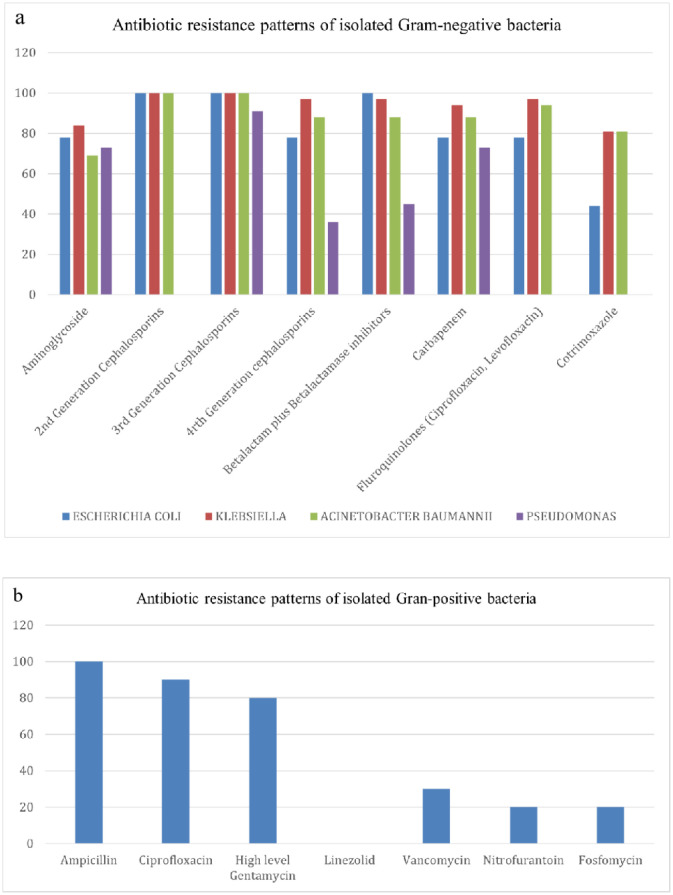
(a) Antibiotic resistance patterns of isolated Gram-negative bacteria; (b) Antibiotic resistance patterns of isolated Gran-positive bacteria.

The antibiotic susceptibility testing data showed that 94% of the *Klebsiella* spp. were resistant to Carbapenem. 88% of *Acinetobacter baumannii* isolated from the ICU patients were Carbapenem-Resistant *Acinetobacter baumannii* (CRAB). Carbapenem resistance was also high among *Escherichia coli* (78%) and *Pseudomonas* spp. (73%) (Figure 3a). Among Gram-positive bacteria, 30% VRE were isolated from the ICU (Figure 3b).

**Figure 4.**
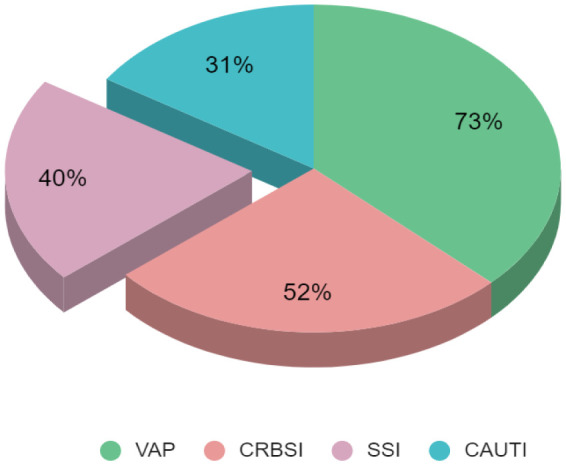
Outcomes in terms of mortality in different types of HAI. CRBSI = Catheter Related Bloodstream Infection; VAP = Ventilator Associated Pneumonia; CAUTI = Catheter Associated Urinary Tract Infections; SSI = surgical site infections.

**Figure 5.**
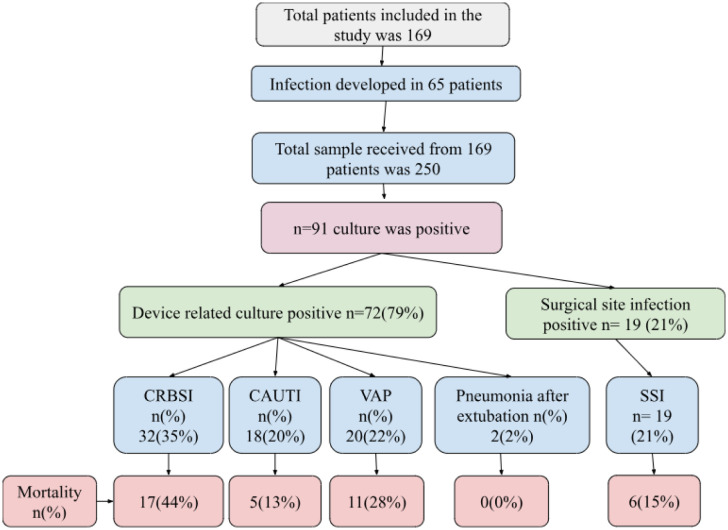
Flowchart showing correlation of culture positivity with patient outcomes in this study.

Figure 4 demonstrates that the highest mortality was associated with VAP (73%), followed by CRBSI (52%), SSI (40%) and CAUTI (31%) in the ICU. The flowchart within Figure 5 shows a summary of the correlation of culture positivity with patient outcomes.

## 4. Discussion

Device related infections in the ICU can be prevented if there is detailed knowledge on the rate of infection, the source of infection, and common causative organisms and their sensitivity patterns [28]. We performed this study to discover the above variables in the ICU to curb the mortality rate in the ICU related to device associated infections. The device associated HAI rate in our ICU was 79%. The presence of an endotracheal tube in the airway causes a breach in natural defence mechanisms such as the cough reflex and impaired mucociliary clearance, which results in micro aspiration. The microorganisms along with the pooled secretions accumulated around the cuff may trickle down the respiratory tract due to the gravity. Additionally, as the tube is a foreign body, there is an increased change of forming a biofilm by the oropharyngeal flora, which subsequently increases the chances of developing VAP [29]–[31].

Among the two types of central line catheters, tunnelled and non-tunnelled, the latter was inserted in all of our patients. According to other studies, the normal skin flora of the patient is responsible for CLABSI within seven days of insertion. The skin flora can migrate towards the intravascular space along the external surface of the inserted catheter. CLABSI that occur within 7 days are due to the migration of pathogens from the skin entry site during catheter insertion. After 7–10 days, MDR bugs found in the hospital environment are responsible for the infection due to contamination of the catheter hub by contaminated hands of health care providers during handling [32].

The most common route of UTIs is the ascending route of infection, where the urogenital commensals adhere and colonise the mucosa of the urethra and then ascend the urethra to reach the bladder. First, urinary catheters provide a direct connection of the environment to the urinary bladder, thus providing a channel for the colonised urogenital flora to ascend to the bladder. Second, the external surface of the catheter provides a suitable site for the microorganisms to form biofilms. Urination flushes out bacteria and other transient microorganisms from the urinary tract, which helps in keeping the urinary tract normally sterile. The presence of the catheter impairs the normal flushing mechanism, and this is the third reason for the predisposition of UTIs in catheterised patients. Fourth, during catheter insertion, there is always minor trauma that occurs in the urothelium where the mucopolysaccharide coating is disrupted, which leads to an immune response and the accumulation of fibrinogen. This helps the uropathogens that express the fibrinogen binding proteinto adhere to the catheter [33].

We included all types of SSIs—superficial incisional infections, deep incisional infections, and organ infections—in our study. The sources of SSIs may be from direct contact or airborne transmission of bugs present in the hospital environment or may be due to endogenous flora [34]. There has not been a study that uncovered an increased incidence of SSIs among critically ill patients. CRBSI was the most common device related to HAIs in our ICU, which contrasts other studies that highlighted VAP was the most common entity. Previous studies found that infections in pediatric ICUs were commonly associated with device insertion, and CLABSI was the most common device associated infection among the study group [35]–[37]. These findings along with the other studies performed on ICU infections corroborates with our study.

In this study, MDR *Klebsiella* spp. was the most common isolated organism from VAP (50%), CRBSI (34%), and SSI (32%), while second most common isolated organism was CAUTI (22%). A study done in Poland showed MDR *Acinetobacter baumannii* as the most common pathogen in VAP and CAUTI, and methicillin-resistant *Staphylococcus epidermidis* (MRSE) as the most common in CLABSI [38]. A recent study showed that among the Gram-negative organisms that cause VAP, *Acinetobacter baumannii* was the common, followed by *K. pneumoniae* [39]. In our study, the most common that cause CAUTI was *Enterococcus* (27%), which is probably due to the ability of this organism to form biofilms which promote its adherence to the catheter [40]. Enterococci are resistant to commonly used antiseptics and disinfectants used in ICUs, so it can remain alive on inanimate objects present in the hospital environment. This is the probable reason for cross-contamination of the bug among patients through the hands of healthcare workers [41]. *Enterococcus* is intrinsically resistant to several antibiotics such as cephalosporins, trimethoprim-sulfamethoxazole, and aminoglycosides. Additionally, it can acquire resistance genes by horizontal genetic transfer and confer resistance to high-level aminoglycoside, ampicillin resistance, and vancomycin resistance [42]. 30% of the *Enterococcus* isolates found in this study were resistant to Vancomycin. The prevalence of VRE in India was found to be 12.4% in a recent study by Smout E, et al. [43]. The VRE rate in the ICU was found to be nearly 68% by Sivaradjy M, et al. [44]. More than 80% of *Klebsiella* isolates in our study were MDR, with carbapenem resistance seen in 84%, aminoglycosides resistance seen in 84%, and fluoroquinolones resistance seen in 97% of the isolates. The antibiotic resistance pattern was corroborated with our previous study [45].

The percentage of MDR *Klebsiella* spp. may vary geographically; this might be due to variations in antibiotic policies, infection control measures in hospitals, and the types of patients served [46]. One hundred acquired genes have been identified in *Klebsiella*, which indicates that horizontal gene transfer is an important factor in developing resistance for these bacteria [47],[48]. According to a review report, carbapenem-resistant *Klebsiella* was responsible for the 48.9% mortality among ICU patients [49]. The WHO has now labeled extended-spectrum β-lactam-producing and carbapenem-resistant *K. pneumoniae* as a critical public health threats [50].

Several studies all over the world have shown that device-related infections in ICUs can be prevented through the use of evidence-based recommendations and prevention bundles. VAP was associated with the highest rate of mortality in our study. This study corroborated the findings of Victor D Rosenthal, et al., who found a higher mortality rate in VAP [51]. However, other studies have shown higher mortality rates in CLABSI cases [52]. Catheter based infections can be reduced by educational programs for healthcare providers that provide training on aseptic methods for catheter insertion techniques. More attention should be given to the exposure time of these devices, and the necessity of the devices should be evaluated daily to reduce the rate of infections [53]. In addition, a stringent cleaning with appropriate disinfectants in ICUs is one of the most cost-effective procedures to reduce ICU infections. Stringent adherence to hand hygiene by healthcare personnel is also an important but easy step in reducing ICU infections by MDR bugs. This work showed the problem with HAIs, and additional studies on this will help to prevent the spread of HAIs.

## 5. Conclusions

HAIs in ICUs include mainly four types of infections: CRBSI, VAP, CAUTI, and SSI. This study demonstrated the antibiotic resistance patterns of common microorganisms found in the ICU, which is an important aspect to improve patient treatment with proper antibiotic regimens. In this study, among 169 patients, 65 patients (38%) were diagnosed with HAIs in the ICU. The most isolated organism in the ICU was *Klebsiella* spp. (35%), followed by *Enterococcus* spp. (22%). Both *Klebsiella* spp. and *Enterococcus* spp. can colonise within admitted patients from the hospital environment. Catheter related infections are preventable if aseptic protocols are followed during insertion. The findings of this study are of great clinical importance and will help in preventing and controlling the spread of HAIs in the ICU.

## Use of AI tools declaration

The authors declare they have not used Artificial Intelligence (AI) tools in the creation of this article.
